# Multimodal Wireless Sensor Network-Based Ambient Assisted Living in Real Homes with Multiple Residents

**DOI:** 10.3390/s140609692

**Published:** 2014-05-30

**Authors:** Can Tunca, Hande Alemdar, Halil Ertan, Ozlem Durmaz Incel, Cem Ersoy

**Affiliations:** 1 NETLAB, Computer Networks Research Laboratory, Department of Computer Engineering, Bogazici University, Bebek, Istanbul 34342, Turkey; E-Mails: hande.alemdar@boun.edu.tr (H.A.); halil.ertan@boun.edu.tr (H.E.); ersoy@boun.edu.tr (C.E.); 2 PeraLab, Pervasive Computing Laboratory, Department of Computer Engineering, Galatasaray University, Ortakoy, Istanbul 34349, Turkey; E-Mail: odincel@gsu.edu.tr

**Keywords:** ambient assisted living, wireless sensor networks, human activity recognition

## Abstract

Human activity recognition and behavior monitoring in a home setting using wireless sensor networks (WSNs) provide a great potential for ambient assisted living (AAL) applications, ranging from health and wellbeing monitoring to resource consumption monitoring. However, due to the limitations of the sensor devices, challenges in wireless communication and the challenges in processing large amounts of sensor data in order to recognize complex human activities, WSN-based AAL systems are not effectively integrated in the home environment. Additionally, given the variety of sensor types and activities, selecting the most suitable set of sensors in the deployment is an important task. In order to investigate and propose solutions to such challenges, we introduce a WSN-based multimodal AAL system compatible for homes with multiple residents. Particularly, we focus on the details of the system architecture, including the challenges of sensor selection, deployment, networking and data collection and provide guidelines for the design and deployment of an effective AAL system. We also present the details of the field study we conducted, using the systems deployed in two different real home environments with multiple residents. With these systems, we are able to collect ambient sensor data from multiple homes. This data can be used to assess the wellbeing of the residents and identify deviations from everyday routines, which may be indicators of health problems. Finally, in order to elaborate on the possible applications of the proposed AAL system and to exemplify directions for processing the collected data, we provide the results of several human activity inference experiments, along with examples on how such results could be interpreted. We believe that the experiences shared in this work will contribute towards accelerating the acceptance of WSN-based AAL systems in the home setting.

## 1. Introduction

Wireless sensor networks (WSNs) are utilized as a promising and efficient solution for various application domains, ranging from environmental monitoring to healthcare, as they provide advantages with their low-cost nature and collaborative sensing/intelligence capabilities. Home automation systems, or smart homes, appear as one of the example application environments where WSNs are utilized. Light control, remote control, smart energy management, security and safety are the example applications that utilize sensing information in the home setting [1]. Furthermore, remote care or ambient assisted living (AAL) applications also exploit WSNs to support the elderly in their daily lives [2] in the context of smart home systems.

The monitoring of wellbeing has become an important challenge for aging societies [3], not only because more investment is needed for elderly care, but also the decrease in the working population causes a shortage of skilled caregivers. The solutions for the continuous and automatic monitoring of wellbeing can contribute toward addressing these challenges. For this reason, developing smart care systems that can enable people to sustain independent living for longer durations and to have a higher quality of life is important. In this regard, WSNs with their smart sensing and communication capabilities are recognized as an ideal platform [2] for AAL environments.

AAL systems utilizing WSNs can offer various services, ranging from fall detection, which is a life-threatening situation for the elderly, to the monitoring of medication intake [4]. One of the important services that can be offered by such a system is remotely assessing the physical and cognitive wellbeing of the people by monitoring the activities of daily living (ADL), such as sleeping, cooking, eating and going out. These activities can be used to characterize human behaviors, and with continuous monitoring, anomalies can be detected early and required actions can be taken quickly.

Although such a system can offer a variety of benefits, there are still challenges in the continuous behavior and wellbeing monitoring using WSN-based AAL systems, stemming from the selection, deployment and the wireless networking of the collaborative sensors. Human activity inference also poses a serious challenge.

The main issues to be considered in sensor selection and deployment are privacy, unobtrusiveness and robustness. To preserve the privacy of the residents, video or sound recording devices are not viable sensing components. The sensors deployed in an AAL system must provide the limited and sufficient information that would enable the recognition of only a certain set of ADL, which we believe are indicative of a person's wellbeing. Any information that could be utilized beyond this purpose would violate privacy and hence should be avoided in an effective system. This approach is also helpful in achieving unobtrusiveness, since an increase in the number of sensors beyond the designated requirements could affect daily activities and, in return, intrude into the residents' daily lives. The usage of wearable sensors, which may disturb and limit the body movements of people, are also not preferable. While achieving these purposes, the robustness of the system should be ensured. The ambient sensors utilized in such a system should blend in with the environment and be able to operate for extended periods of time without any errors. The physical integrity of the sensor devices should not be easily affected by the typical activities of the interacting users. Another challenge is to choose the correct type of sensors targeted to the recognition of specific activities, which is closely related to the sensor characteristics and the user preferences.

From the perspective of wireless networking, the home is a typical multi-path environment [1], due to the walls, furniture and people moving around, and it is subject to interference, since ISM (industrial, scientific and medical) bands, which are usually used by WSNs for communication, are heavily utilized by cordless phones, Bluetooth and WiFi devices and even microwave ovens. Still, sensors operate using battery power, and the network lifetime is limited. Besides the battery limitations, sensor resources are limited in terms of processing, communication and storage. Therefore, they cannot operate complex software and perform complex calculations.

One of the fundamental requirements of an AAL system is the recognition of human activities automatically. For a system composed of ambient sensors, the primary challenge is identifying human activities solely from the sensor data generated by the influence of human interactions of varying degrees. The nature of human activities are usually very complex: multiple tasks can be performed concurrently, and an activity can be performed in different ways, sometimes with a different sequential ordering. Overall, these characteristics impact the activity recognition performance negatively. Usually, statistical machine learning techniques are used to infer activities from the sensor data. In this approach, finding an accurate inference model poses a significant challenge. Furthermore, training data sets that contain the ground-truth labels of the activity data together with the raw sensor data are required. Obtaining these labels requires human involvement, for instance the user may be asked to keep a diary or, using an automatic voice recognition system, the activities can be automatically labeled. Labeling the data in the training phase is a tedious and costly process; thus, publicly available data sets that are collected in realistic conditions are extremely important [5].

**Figure 1.**
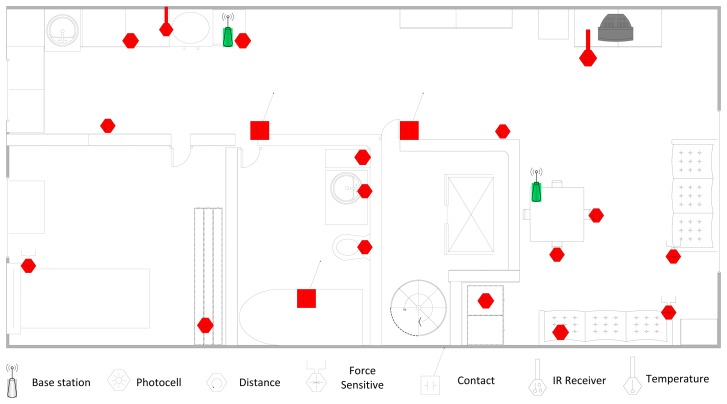
Layout and sensor deployment of House A.

**Figure 2.**
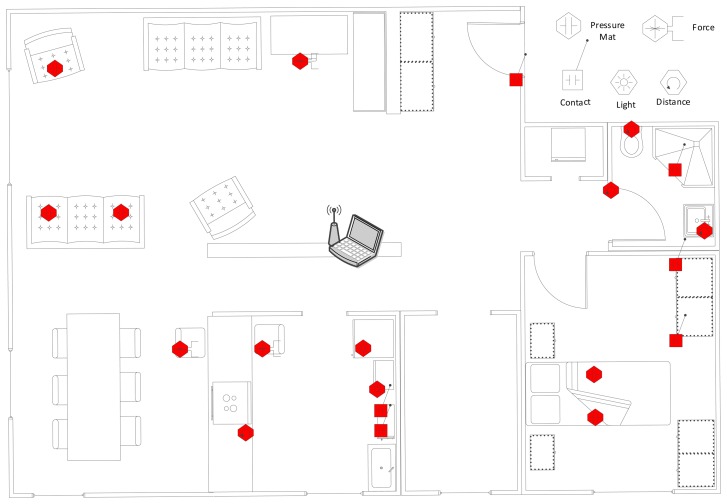
Layout and sensor deployment of House B.

In order to address these challenges, we propose a WSN-based automated multimodal AAL system compatible with homes with multiple residents, which has been tested by a field study in two real houses, each shared by two residents. For each house, we deployed 20 sensors of various types and collected ADL data consisting of sensor readings for 30 full days. The layouts of the houses together with the type and location of the deployed sensors are shown in Figures 1 and 2. We have utilized the Arduino platform [6], specifically battery-powered Arduino Fio motes, as the sensor hardware and used ambient sensors, including force sensitive resistors (FSR), photocells, digital distance sensors, sonar distance sensors, contact sensors, temperature sensors, pressure mats and infrared receivers. The motes equipped with Xbee modules have the capability of establishing WSNs by wireless communication using the ZigBee protocol.

To establish a framework enabling the collection of annotated data with activity ground-truth labels, we have designed a simple activity labeling interface to be used by the residents. Twenty seven different activities could be recognized by the system, including the main daily living activities, like sleeping, toileting, having a meal, cooking, watching TV, taking a shower and activities that are not performed every day, like hanging out laundry, doing cleaning and having a nap. In this work, to shed light on the possible monitoring and recognition capabilities of the proposed system, we also include the inference results of five different classifiers, namely k-nearest neighbors, decision tree, hidden Markov model, multi-layer perceptron and time-delayed neural network, for ADL recognition purposes.

We list the main contributions and the highlights of our work as follows:

- Compared to the previous examples of AAL systems, which were deployed in a laboratory instead of a real house or considering only a single resident and focusing on a limited set of activities, our system was deployed in two real houses, each shared by two residents, identifying a detailed set of 27 activities for 30 full days.
- We present the conducted field tests of our system while summarizing our experiences in meeting the challenges related to sensor selection, deployment, wireless networking, autonomous system operability and robustness, with the intention of contributing to future studies on the acceptance and commercialization of WSN-based AAL systems. We also define and present specific sensor selection criteria, including an analysis on the convenience of specific types of sensors in terms of robustness, efficiency and targeted activity in house environments. We believe that such an effort will guide the readership for the future deployment of similar systems.
- We investigate the performance of the battery lifetime when ambient sensors are duty cycled and transmit event-based data and show that the battery lifetime can significantly be improved compared to high-rate sampling and transmission.
- To provide insight into the applicability of the proposed system for monitoring and human activity recognition purposes, we discuss the possible directions for processing the data collected by the system and present the results of the activity inference experiments, conducted using five different machine learning models of varying complexities and characteristics.

The primary scope of this work is the design of a low-cost, efficient and realizable AAL system targeted for real houses, with a focus on the sensor selection/deployment criteria, system description and activity recognition applications.

The organization of the paper is as follows: In Section 2, the literature review on AAL systems based on WSNs is presented. In Section 3, we introduce the design and architecture of the proposed AAL system. In Section 4, the details of the field study conducted in two real homes are presented. In Section 5, we elaborate on the possible applications of the proposed system and, as an example, share the results of the human activity inference experiments conducted on the data collected by the field study. Finally, Section 6 concludes our study and provides possible directions for future research.

## 2. Related Work

In a very recent study [7], a literature survey of state-of-the-art AAL frameworks, systems and platforms to identify the essential aspects of AAL systems was provided. Smart home monitoring systems that target assisted living can have different objectives. While some of them may support the elderly for the prevention of dangerous situations, such as falls [8], some focus on health issues, such as monitoring medication intake [4]. In this part, we specifically elaborate on the smart home AAL systems that aim at monitoring the activities of the elderly.

In [9], a smart home monitoring application for assisted living was introduced. The system monitors the use of electronic appliances with current sensors, the water usage with water flow sensors and the bed usage using a force sensor for determining the sleeping pattern of the elderly. The collected data is transmitted to a central server, and if abnormal situations occur, such as excessive water usage, the system informs the related people by sending an SMS (short messaging service) message. A prototype of the system was deployed in a two-bedroom house with six sensors. However, no performance results were presented in the paper. Similarly, in [10], well-being conditions of the elderly based on the usage of house-hold appliances are monitored using ZigBee-based wireless sensors. Current sensors monitor the use of electric appliances, force sensors were attached to the bed, couch, toilet and dining chair to monitor their daily usage and contact sensors were attached to the grooming cabinet and fridge to monitor the opening and closing of the doors. Two wellness functions are defined according to the use of house appliances and their inactivity. The system was deployed in four houses with six sensors for a week and collected data in real time about the wellness of the elderly. In an alternative system [11], instead of monitoring the daily activities of the elderly, acceleration sensors were attached to sanding hand blocks for monitoring the exercise routines of the elderly. A similar system that utilizes body sensor networks for physical movement monitoring of the elderly was presented in [12].

Supervised machine learning methods for human activity recognition using WSNs have also been investigated in the literature [13–17]. In [13], reed switches and piezoelectric switches were installed in two different homes on doors, windows, cabinets, drawers, microwave ovens, refrigerators, stoves, sinks, toilets, showers, light switches, lamps, some containers and electric/electronic appliances to detect more than 20 activities. The collected data was labeled by the subjects using software running on a PDA (personal digital assistant), was processed using a naive Bayes classifier and revealed a performance of 25% to 89%, depending on the evaluation metric used.

In [14], van Kasteren *et al.* (2008) deployed a WSN-based system consisting of 14 sensors in a real house and collected data for 28 days. The data was automatically labeled by the subject using a Bluetooth headset with voice recognition software. The deployment targeted the classification of seven activities, and the data was processed using both the hidden Markov model (HMM) and conditional random fields (CRFs). They reported an accuracy of 79.4%.

In [15], 15 different activities were monitored using a smart home testbed, which was equipped with motion and temperature sensors, as well as analog sensors that monitor water and stove burner use. The system was tested in a multi-resident environment, where two students lived together. The data was processed using an HMM. In the first evaluation, they put all of the sensor data for the 15 activities into one dataset, and the HMM revealed 60.60% accuracy. In the second evaluation, they generated one HMM for each resident, assuming that they knew the person performing the activities for each event. In this case, the average accuracy was computed as 73.15%.

In another recent study [16], the use of hybrid models was proposed for increasing the accuracy of activity recognition. The authors combined the artificial neural network (ANN), specifically multi-layer perceptron (MLP) and support vector machine (SVM), with HMM and show that hybrid models achieve better recognition performance compared to MLP, SVM, decision tree (DT), k-nearest neighbors (kNN) and a rule-based classifier. They used five different datasets, including three datasets from [18] and two datasets collected by the authors that included 12 different activities.

Fatima *et al.* (2013) take it one step further and proposed a unified framework for action prediction besides activity recognition in [17]. An SVM-based kernel fusion method was utilized for activity recognition and identifying the significant sequential activities of the inhabitants to predict the future actions. CRF was used as a classifier for predicting the future actions. They used the CASAS (Center for Advanced Studies in Adaptive Systems) datasets presented in [19]. The performance of the kernel fusion method was compared with other kernel methods, including linear kernel, radial basis function (RBF) kernel, polynomial kernel and MLP kernel, and it was shown that a 13.82% increase is achieved in the accuracy on average for recognized activities. For action prediction, the performance of CRF was compared with HMM, and it was shown that an increase of 6.61% to 6.76% is achieved in the F-measure with CRF.

While most of the previous work focused on achieving higher physical activity recognition performance with indoor or outdoor applications using different sets of sensors, research trends are moving towards human behavior understanding, such that the habits and daily routines of people are discovered and the causes of drifts from these patterns are analyzed [20]. Compared to the discussed systems, we first present a guideline on sensor selection for human activity recognition with a WSN-based AAL system considering different criteria, including the targeted activity, robustness and efficiency of the sensor types. Different than the previous work, our system utilizes both a higher number and a higher variety of sensors (20 sensors with seven different types) targeting a large number of more complex activities, *i.e.*, 27 different activities can be identified, and the system collected data for a longer interval of 30 days. While most of the previous studies only considered the recognition of the activities of a single resident, in our work, we investigate the detection of activities of multiple residents, since it is not unusual that the elderly do not live alone in a house. Moreover, in our system, we do not assume that the person IDs for the activities are known, in contrast to the study in [15], which also targets multiple residents, which is a more challenging problem compared to most of the similar studies. Additionally, instead of utilizing a single classifier, we evaluate the performance of different classifiers in identifying the activities in a multi-resident setting.

## 3. System Architecture of Multimodal WSN for AAL

In this section, we present the architectural details of the proposed WSN-based AAL system. We also explain the challenges related to the sensor selection/deployment, networking and data collection and present the respective solutions that we have devised. With such an approach, we aim to provide design criteria and guidelines for different components of multimodal WSN-based AAL systems, with the intention of also assisting future research. The Arduino platform together with the Xbee transceiver modules, which use the ZigBee protocol, are used to enable the sensing and wireless communication components. The Arduino platform is an open source, cost- and power-efficient hardware platform, which helped us to quickly prototype the different sensor modalities that we required for the AAL system.

### 3.1. Sensor Selection and Deployment

In the scope of this work, we have deduced several criteria for the selection of different sensor types suitable for AAL applications. These criteria guided us through both the general and activity-specific sensor selection and deployment decisions and allowed us to overcome challenges related to the robustness and efficiency of the individual sensors and the overall system.

The foremost decision regarding the use of ambient sensors rather than wearable sensors stems from the possible concerns of the potential system users regarding obtrusiveness. Wearable sensors that are directly attached to the body or clothes are not a viable choice, since they may be uncomfortable, intrusive and even limit the bodily movements of the users. Privacy is also a significant concern that we addressed. We avoided the use of cameras, video recorders or microphones, since such devices pose a direct threat to the daily life privacy of the users. These strict guidelines ensure that the proposed AAL system is privacy-preserving and unobtrusive.

The ambient sensor devices available in our inventory include force sensitive resistors (FSRs), photocells, digital distance sensors, sonar distance sensors, contact sensors, temperature sensors, infrared receivers and humidity sensors. FSRs produce readings inversely proportional to the changing resistance according to the force applied to it. The photocells are sensitive to the change of the amount of light in the environment. Digital distance sensors measure object presence in small ranges within 10 cm. Sonar distance sensors can measure the presence of objects at higher distances, up to 6.5 m. Contact sensors produce readings according to the contact of their two separable components. Temperature sensors measure the environmental temperature. The humidity sensors measure the relative humidity of the environment.

The specific choice among the different types of ambient sensors is influenced by three primary criteria: targeted activity, robustness and efficiency. To assess the performance of different sensors with respect to these criteria, we have conducted experimental dry runs with the individual sensor devices under various activity scenarios before the actual deployment of the system. Moreover, we interviewed the residents about the usage of the goods and items at their homes to assess their individual interaction patterns. Such interviews enabled us to make more accurate decisions on the type and deployment location of the sensors and to better match particular activities with the sensors. The performance and convenience of the specific types of sensors with respect to the above-mentioned criteria are summarized in Table 1. Next, we present details about these criteria and provide example scenarios in an attempt to heighten the understanding level of the readership.

#### 3.1.1. Targeted Activity

Since the proposed AAL system's ultimate aim is to recognize the activities of the residents using the data coming from the deployed sensors, we primarily decided on the sensor types and their locations by matching them with the targeted activities. For instance, during the teeth brushing activity, we expect several actions to occur at the same time or in succession. For instance, to recognize this activity, we might deploy a contact sensor on the bathroom door, a photocell in the bathroom cupboard and a digital distance sensor above the water tap. The teeth brushing activity is expected to be performed as follows. Firstly, the person closes the bathroom door, which triggers the contact sensor, which is located on the side of bathroom door. It continues firing during the activity, since we expect the bathroom door to be closed during the activity. After a while, we expect the photocell located in the cupboard to be activated when the subject opens the cupboard door to get the toothbrush. As the cupboard door is closed, we expect the photocell to stop firing. After the person has finished brushing his/her teeth, we expect the digital distance sensor above the water tap to fire for a short time when the subject is washing his/her mouth. Finally, we expect the contact sensor at the bathroom door to stop firing as the bathroom door is opened and the teeth brushing activity ends. It should be noted that the sensor selection for this scenario is done based on our intuitive belief on the succession of actions related to this targeted activity. To further increase the compatibility of the sensors to the targeted activities, we conducted short interviews with the residents to assess the ways they perform the targeted activities. For instance, they stated that they always keep the toothbrushes in the bathroom cupboard and keep the bathroom door closed when brushing their teeth. Even though such interviews do not form the basis of our sensor selection criteria, they have definitely been helpful for choosing the adequate types and deployment locations of the sensors.

#### 3.1.2. Robustness

The sensors should be selected so as to keep the components of the sensor devices intact and to allow them to function without malfunctioning in the event of possible activities involving the interaction of the users with the sensors. For instance, we have initially used an FSR sensor, which is placed under the leg of a chair, to detect the action of sitting on the chair. However, it was not sufficiently robust, even if we placed it in a stable position, because it was in contact with the ground, and since the chair is a mobile item, it had a high probability of breaking down or coming apart during the operation of the system. Instead, we preferred to use a digital distance sensor located at the back of the chair to detect the sitting in the chair action. The properties of the specific chair in that house also influenced this decision, since the back of the chair had an appropriate hole to accommodate such a sensor. As another example, we can give the toilet flushing action. Initially, we have used FSR and contact sensors positioned on the flush button successively. However, they were not robust enough to give consistent results. They were prone to dislocation by the physical contact of the users. Therefore, we placed a digital distance sensor to the toilet seat cover to recognize whether it is open or not. As a learned lesson, we can state that the sensors should be selected and deployed so as to minimize the contact between the sensors and subjects, in order to increase robustness, hence enabling clean and consistent results.

#### 3.1.3. Efficiency

No matter how robust or intuitively convenient a sensor is for a targeted activity, it cannot be considered an adequate choice unless it is efficient. Efficiency is directly related to the correctness and completeness of the readings a sensor generates in harmony with the targeted activity. As an example, we have initially used the humidity sensor to detect the activity of taking a shower. However, using this sensor, the exact duration of the showering behavior could not be inferred, since the relative humidity in the bathroom does not decrease rapidly; hence, the humidity sensor continues to give high humidity values for a long time even after the activity is completed. Therefore, we decided to use a contact sensor located on the shower cabin door to detect whether the shower cabin is closed. Another example is the choice between the photocell and the vibration sensors to detect if a drawer is opened. The vibration sensor placed inside a drawer starts firing with the motion of opening the drawer, as expected; however, even after the drawer is closed, the vibrations continue, thus making it impossible to infer when the action ends. Therefore, we preferred photocells that are far more efficient to detect such an action. The tuning of the threshold values for the initiation of sensor firings also plays an important role in adjusting and enhancing the sensor's efficiency. For instance, to detect if a person is sleeping, we were able to use an FSR sensor, since setting a threshold enabled the sensor to recognize the extra weight of the person in addition to the weight of the bed. However, the sensitivity of a specific sensor ultimately determines if such a fine threshold could be set; hence, the efficiency of a sensor also depends on its sensitivity with respect to the sensing requirements of a targeted activity.

**Figure 3.**
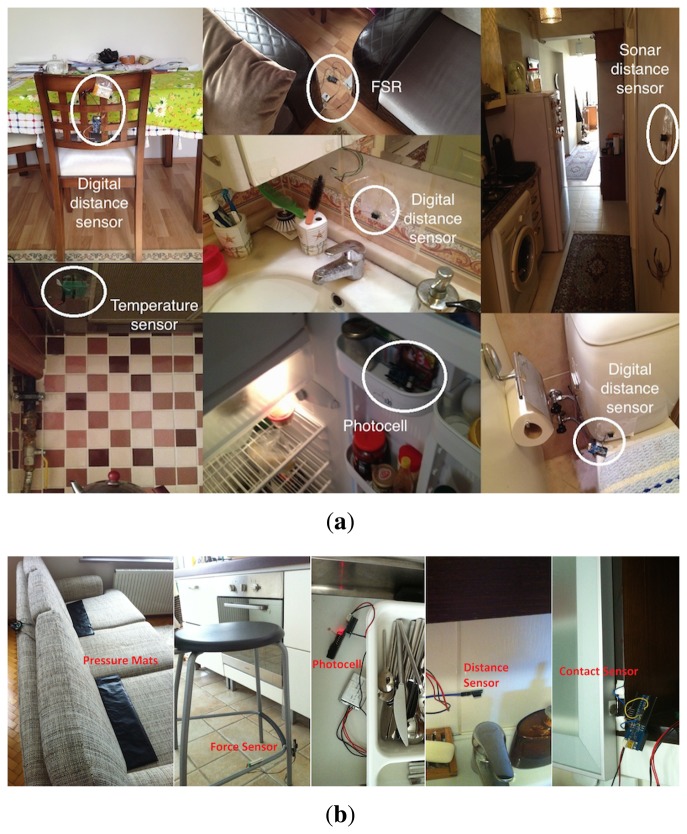
Example deployments of ambient sensors considering the designated criteria. (**a**) House A; (**b**) House B.

Several example sensor deployments in real houses, which are made considering the above-mentioned criteria, are shown in Figure 3. The actual sensor deployments for the field study performed in two real houses are provided in Section 4.

#### 3.2 Networking

The proposed system's networking component is composed of star-topology ZigBee (IEEE 802.15.4) networks. Depending on the coverage of the central base stations (coordinators), the network consists of one or a few personal area networks (PANs) operating in different channels. In case of multiple PANs, the coordinators (clusterheads) of individual PANs communicate with each other through a base station (e.g., access point), hence creating a star-star tree topology. We use commercialized Xbee transceivers, compatible with the Arduino modules, as the ZigBee solution. Due to the obstacles and walls affecting the signal propagation in a typical house, multiple PAN coordinators may be required to achieve complete coverage of the deployed sensor devices. The PAN coordinators should be deployed to provide line-of-sight communication with as many sensors as possible. The communication channel selections are to be made based on their overlap with the WiFi networks in the vicinity, since ZigBee and WiFi standards utilize overlapping bandwidths (2.4 GHz ISM band).

Each sensor unit sends sensor values to the associated PAN coordinator when an event is detected. The sensors and coordinator within the same PAN utilize the same channel for transmissions, which is to be set differently from the channel's other PANs use, in order to prevent interference. The PAN coordinators are connected to a central processing unit via a serial interface. In the central unit, the data from the two subnetworks and the ground-truth labels are matched and synchronized, for which the details are given in the next section.

The sensor nodes are configured to transmit data in an event-based binary format, although the sensors being used are not binary. The sensors produce values from 0 to 1024. In order to convert the sensor data to binary format, we use thresholding. During the operation of the system, a sensor is sampled 10 times in a second, and the sensor value is compared with the predefined threshold value specific for that sensor. If the sensor value exceeds a predefined non-activity range, an event is detected. Upon detection, the sensor node wakes up its transmitter and starts transmitting binary data to the relevant PAN coordinator. As the sensor values fall back under the specified thresholds, data transmission stops, and the transmitters are switched to sleep mode again.

In order to determine the effect of different transceiver duty cycling and sampling rates on the sensor battery depletion durations, we conducted several experiments before the deployment. Extending the lifetime of the sensor batteries and, hence, the whole WSN is of utmost importance, since this directly affects the system's capability to operate autonomously without the need for frequent human intervention. In the experiments, we used 1800 mAh lithium-polymer batteries. In the transmission mode; the current consumption is 45 mA, while in the idle/reception mode, it is 50 mA. In sleep mode, the current consumption is below 10 μA. The results are summarized in Table 2.

According to the results, we obtain approximately one day of lifetime when duty cycling (sleep mode) is not utilized. The effect of the sampling rate is negligible. There is not much improvement with lower sampling rates, since the sensors being used have much lower power consumption compared to the radio, which is consistent with the specifications of the hardware. On the other hand, when we use duty cycling, we see improvements in the battery lifetime, as expected. Surprisingly, the impact of sampling rate on the battery lifetime also increases as the duty cycle decreases. Regarding the tuning of the sampling rate, we observed that higher sampling rates do not provide any extra information considering the durations and the nature of the human activities. Therefore, we preferred using a 10-Hz sampling rate to achieve a prolonged battery lifetime.

Despite the increased battery lifetime advantage of duty cycling, it has a notable drawback. Although the wake-up time for the Xbee module is 13.2 ms, which is quite a low value, the reassociation with the PAN coordinators can take as long as 300 ms. On the other hand, given the considerable increase in the battery lifetime and the typical duration characteristics of human activities, this delay does not affect the performance of the system significantly; hence, using duty cycling is more preferable. During our 30-day field study, in each of the two houses, the battery replacement frequency varied between two times to eight times, depending on how frequently a specific sensor detects an event and transmit readings.

### 3.3. Data Collection

The sensor data flowing to the PAN coordinators are synchronized and time-stamped at a central component. The raw data obtained in this stage has a granularity of seconds. Since most human activities can be defined in minutes rather than seconds, we use a time-step discretization of one minute.

**Figure 4.**
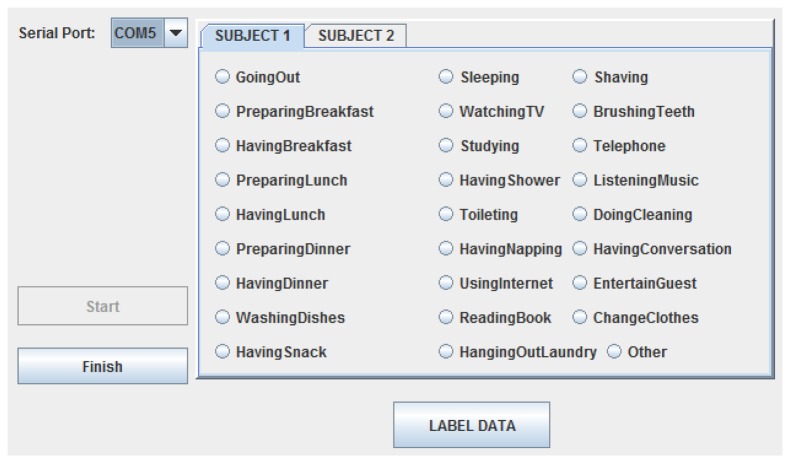
The interface for labeling the activities performed by the residents.

For the purposes of training the machine learning module that would enable the inference of human activities, activity ground-truth labels need to be obtained. Since privacy is of utmost concern for the proposed system, we avoid the use of video cameras. Previous studies use methods, like keeping a diary or using Bluetooth headsets, for annotation. Instead, we provide a software application, running on a laptop situated in the house, with a simple user interface (Figure 4) and ask the residents to provide the ground-truth labels of the activities in which they were engaged. Our method is more accurate than manually keeping a diary and more user-friendly than wearing a headset all the time [14]. Furthermore, we did not ask them to carry any identification sensors on them to ensure unobtrusiveness. Likewise, as previously stated, sensors were placed in convenient locations to ensure the natural behavior of the residents and not to disturb their daily routines. Moreover, during the field study, the residents were not required to follow a specific scenario and were asked to continue leading their daily lives as if the AAL system did not exist. In our field study, there are from 60 to 100 labels for each day, which indicates the level of detail of the ground-truth labels made possible by the user-friendliness of the ground-truth labeling interface. It should be noted that the ground-truth labels need only be obtained for a specific amount of time to be used for machine learning module training purposes. After such a period, the system is expected to autonomously operate without requiring any input from the residents.

**Figure 5.**
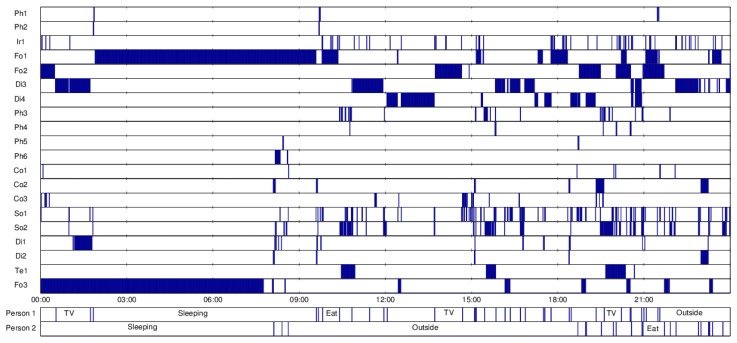
Example sensor readings matched with true activities (24 h).

The labels provided by the residents are also synchronized with the sensor data. As an example, the synchronized sensor data together with the obtained activity labels, for one full day, in one house, are depicted in Figure 5.

**Figure 6.**
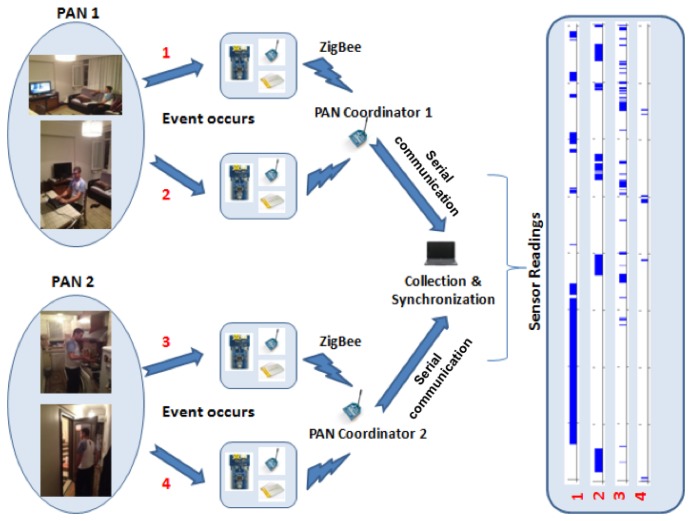
System information flow.

The information flow in a system, deployed according to the design criteria specified in this section, with two PAN coordinators, is demonstrated in Figure 6. The sensors residing in both PANs can pick up simultaneous events (sensor readings exceed a pre-determined threshold), which triggers them to wake their transmitters up and send binary data to the PAN coordinators. The PAN coordinators then relay the sensor data to a collection and synchronization center that aggregates it along with the ground-truth labels provided by the residents.

In the next section, we present the details of the actual systems deployed in two real homes as part of a field study and provide information about the real data collected in a 30-day period. It should be noted that the design criteria specified in this section influenced the entirety of the design and deployment of these actual systems.

## 4. Collection of Real Life Data from Two Homes with Multiple Residents

As a field study, we deployed the proposed system in two real home settings and collected fully-labeled 30 day-long datasets. The details about the two houses (annotated as House A and B), the deployed systems, the residents and the collected data are given in Table 3.

Unlike most of the other similar studies that include the deployment of systems collecting daily living data regarding people, the data we collected from each house is composed of sensor readings influenced by two residents who share the same house. We think that such a setting reflects real life more closely by accounting for most of the people who live with their family, spouse and friends, and additionally, it will give the opportunity to investigate the relationship between couples. Moreover, since we have used real homes instead of controlled laboratory environments and allowed the residents to pursue their normal daily lives and perform their regular behaviors/routines, we believe that the data we collected is highly realistic.

Regarding the activity labeling for ground-truth collection purposes, overall, we could manage to obtain from 60 to 100 activity labels for each day. Twenty seven different activities were captured, including every day activities, like sleeping, brushing teeth, watching TV, toileting, preparing a meal and eating. Rare activities that are not performed every day, such as hanging out laundry, having a guest, doing cleaning and having a nap, are also captured. These rare activities might have great significance in an application inferring the health status or wellbeing of the residents. Therefore, we gathered information about such activities, unlike most of the previous studies.

The total number of activity occurrences in House A and House B is 2177 and 1023, respectively. The total number of sensor readings is around 26 million.

The detailed layouts of Houses A and B along with the locations of the deployed sensors are presented in Figures 1 and 2, respectively. The availability of different sensors for each house with their types and detailed locations are also listed in Table 4.

## 5. Example Application: Human Activity and Behavior Pattern Inference

In this section, to provide insights into the AAL applications of the proposed WSN-based system, we demonstrate several examples of valuable information that is possible to extract from the system. We also show how this information can be obtained using several machine learning algorithms and demonstrate their performance in recognizing the activities of daily living.

### 5.1. What Can Data Tell us about the Residents?

Continuous monitoring of the daily activities of the residents gives strong cues about their behavior and habits. Once the habits are learned, the drifts in the usual behavior that can indicate health-related problems are identified easily. Thus, several precautions are taken earlier and independent and healthy living is sustained for more years. Additionally, every change in behavior is not necessarily a sign of health degradation. For example, a person who has a sedentary life can be encouraged to have more physical activity, and the feedback on the progress can be given to him for a better quality of life.

Ambient assisted living systems imitate or aid the human attendants in smart homes. If we realize that the resident of the house started going to the toilet very often, this may indicate a urinary infection, and we may initiate a urinary sample inspection for further diagnosis. After learning that, normally, the resident uses the toilet a certain number of times during the day, a significant deviation from that frequency can be used for triggering a request for further diagnosis.

Similarly, most of the simple everyday tasks, like preparation of coffee, cooking, reading a book and medication intake, require considerable cognitive skills. Not only major neurological problems, like Alzheimer's disease, but even mild cognitive impairments can be realized. Since our system is identifying these types of activities, we can also follow their durations and frequencies and again use significant deviations from daily averages for further investigation.

**Figure 7.**
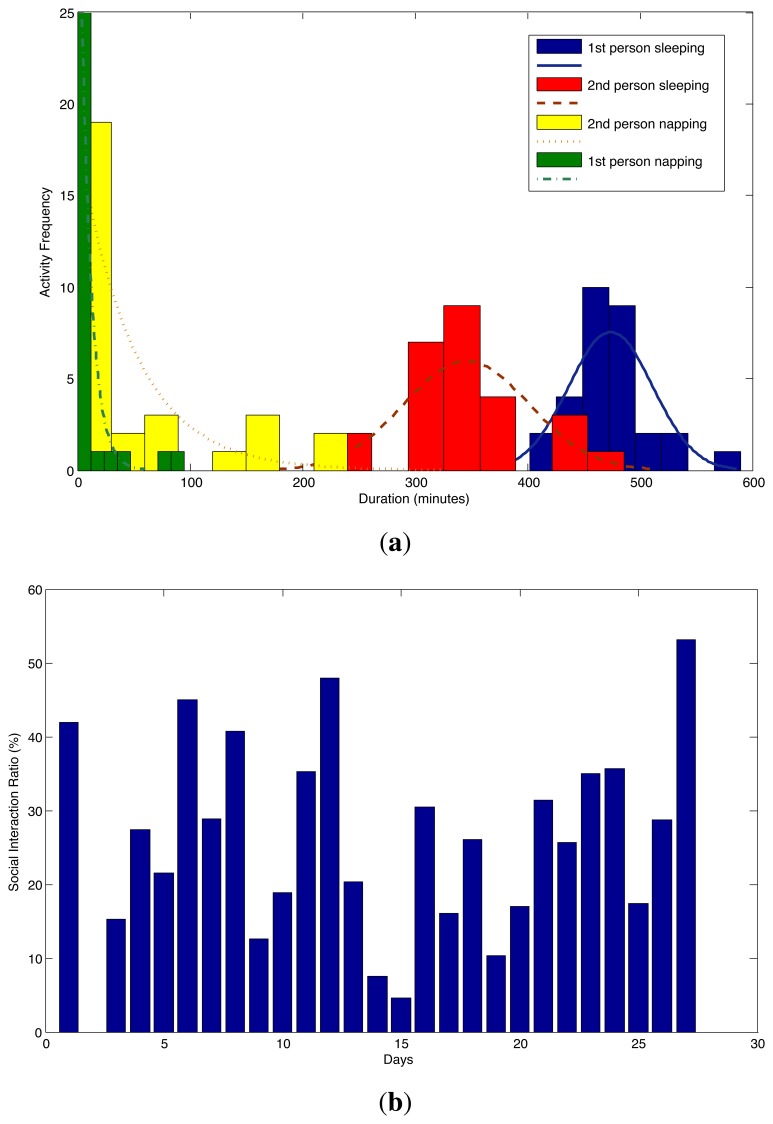
Activity and social interaction behavior patterns. (**a**) Sleeping and napping duration distributions of residents; (**b**) social interaction ratio of time spent together at home.

Human behavior and habits are characterized by three attributes of daily activities, namely the time, duration and frequency. The drifts in behavior can be identified by looking at the changes in those attributes. For example, take sleeping and napping behaviors of persons during a day. Any subtle change in sleeping or napping durations can be a sign of a serious disease, especially for the elderly, or an indicator in the progress of a mental disease, like Alzheimer's disease, in the long term. In Figure 7a, sleeping and napping duration distributions for both residents are depicted. It is directly visible from the graph that the first person sleeps more than the second person. On the other hand, the second person has more episodes of napping. In total, the sleep duration for both residents are within the normal ranges, but their behaviors are completely different. For this reason, having a personalized behavior monitoring system that is capable of learning individual behavior, although there are multiple residents, is of significant importance.

On the one hand, the individual identification of behavior is important; on the other hand, the activities performed together by all residents provide strong clues about the quality of mental health. For instance, having guests in the house, or having a conversation with each other or having a meal together are important activities in terms of mental health. If a person spends most of the time at home having guests very rarely, it can be a sign of depression. In Figure 7b, we depict the ratio of the time (as a percentage) the persons spend together to the amount of time they stay at home. The time spent together includes the durations of having conversations with each other and the durations of common activities performed by the two persons together, like preparing a meal, having a meal, having a conversation, watching TV, except sleeping and going outside together. The average ratio of interacting with each other was calculated as 25.6%. In the long-term monitoring scenario, the changes in this ratio can be used in assessing the quality of social and mental life.

**Figure 8.**
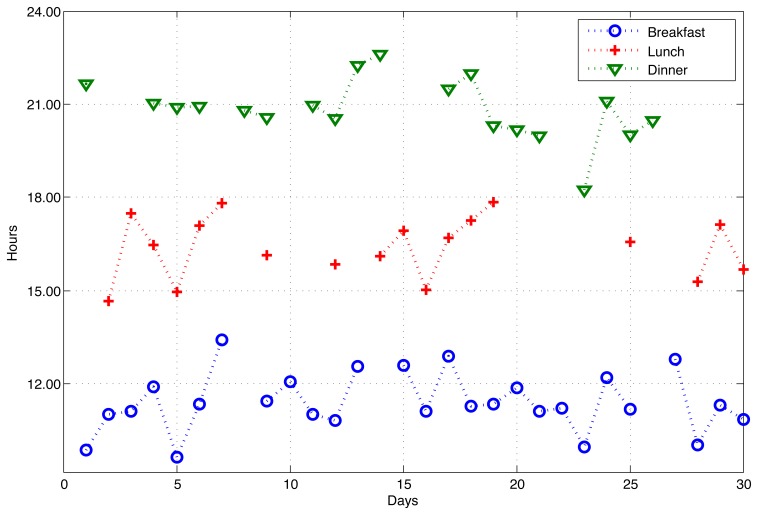
Starting times of breakfast, lunch and dinner of a resident through 30 days.

Other than durations, for some daily activities, the occurrence time is important. In Figure 8, we depict the variations in the starting times of having main meals for a full month. It is evident from the figure that the person showed some unhealthy behavior during that month, such as having breakfast at 1 pm, lunch at 6 pm and dinner at 11 pm. Furthermore, he skipped some meals completely from time to time. Yet, this unhealthy behavior is not recurrent and can be considered as normal. However, if skipping a meal becomes more recurrent, it can be an indicator of stomach-related problems, as well as dementia, and should be taken seriously.

Besides long-term monitoring benefits, the WSN-based AAL systems can provide alerting capability for emergency conditions. To give an example, prolonged bathroom use, say for a half an hour or more, can be a sign of an emergency case for most people. The person can become unconscious after a fall followed by a head injury in the bathroom, for example. That kind of situation is not very rare, especially for the elderly. Such emergency conditions can be detected with the WSN-based AAL system, and healthcare professionals can be informed immediately.

Finally, AAL systems can be deployed to monitor, control and reduce domestic energy and resource consumption. For example, instances of leaving the television on while the residents are elsewhere, such as taking a shower or sleeping, can be detected. The system then either reports the potential reductions in the energy consumption to the residents or directly takes actions to accomplish the reduction, by turning off the television, for instance. Similar scenarios are possible for water usage and air conditioning.

### 5.2. How Can Activities Be Inferred?

To shed light on the possible methods for the recognition of human activities, we conducted preliminary activity inference experiments. Various classifiers are utilized on the datasets collected by the systems deployed in the two previously mentioned houses. In this section, we first describe the experimental setup used in evaluating the performance of our activity recognition system. We then provide the results of the experiments and show the performance of the different classifiers used in our activity recognition system in terms of several metrics.

The activities targeted by the inference system is based on the grouping of similar activities into more general classes, as given in Table 5. Since there are two residents in each house, each performing simultaneous activities, two element power-sets of these activity classes are considered and recognized. In case of overlapping/simultaneous activities, the dominant activity is considered.

In the experiments, we used five different classifiers, namely, kNN, DT, HMM, MLP and time-delay neural network (TDNN). The set of methods we selected have different complexity levels and are from different families of models, *i.e.*, generative and discriminative. kNN is one of the simplest machine learning methods, and it classifies samples based on the majority voting among their k closest neighbors. DTs are one of the widely used models in several classification problems. They are mostly preferred because they can produce good results even when there is a limited amount of training data, and they provide an easier interpretation of the classification rules. HMM is a generative model that is widely used in natural language processing, and also, it has been shown to work well for human activity recognition tasks, because of its capability to grasp the temporal nature of human activities. MLPs are artificial neural networks that are capable of learning a wide variety of problems, including human activities. TDNN is an artificial neural network capable of considering the sequential nature of a time series and, hence, is suitable for processing sequential multimodal sensor data. All of these classifiers are supervised machine learning methods; hence, they require training with the activity ground-truth, which is available thanks to the activity labeling component used during the data collection.

In order to evaluate the activity recognition performance of the classifiers, they are trained and tested with different portions of the datasets. We use the leave-one-day-out cross-validation methodology in our experiments. For each house, we use one full day of data for testing and the remaining days for training. We cycle over the days and use each and every day once for testing. In our case, leave-one-day-out cross-validation is equal to the 30-fold cross-validation.

For measuring the performance, we use precision, recall, the F-measure and accuracy metrics. For precision, recall and the F-measure, we calculate the values for each class separately and then average them over all classes. This is important when the distribution of classes are not balanced, as in the case of human activity recognition. We also report the accuracy, which represents the percentage of correctly classified data points.

(1) $$\text{Precision}=\frac{1}{Q}\sum_{i=1}^{Q}\frac{TP_{i}}{TP_{i}+FP_{i}}$$

(2) $$\text{Recall}=\frac{1}{Q}\sum_{i=1}^{Q}\frac{TP_{i}}{TP_{i}+FN_{i}}$$

(3) $$F-\text{measure}=\frac{2.\text{Precision}.\text{Recall}}{\text{Precision}+\text{Recall}}$$

(4) $$\text{Accuracy}=\frac{\overset{Q}{\underset{i=1}{\text{∑}}}TP_{i}}{\text{Total Number of Data Points}}$$

where *Q* is the number of classes, *TP_i_* is the number of true positive classifications for class *i*, *FP_i_* is the number of false positive classifications for class *i* and *FN_i_* is the number of false negative classifications for class *i*.

We developed an experimentation framework software using MATLAB (Mathworks, Natick, MA, USA). Optimal parameters for classification methods are determined empirically using a validation set. For the kNN classifier, the number of nearest neighbors considered, *k*, is chosen as 80. The Euclidean distance metric is used as the criterion for determining the nearest neighbors in the training set. Among the *k* neighbors, majority voting is used to make predictions about the test samples. A binary decision tree is used for the DT classifier with two children nodes originating from the branch nodes. The splitting decisions are made according to Gini's diversity index criterion. Branch nodes are selected so that they contain at least 100 observations satisfying the inherent rule. For the MLP classifier, a single hidden layer model with 12 hidden units is constructed. The backpropagation algorithm is used for training, performing 20 epochs over the training sets with the learning factor of 0.01. TDNN uses the same parameters and training method as MLP with the additional parameter of the number of delays selected as 10.

**Figure 9.**
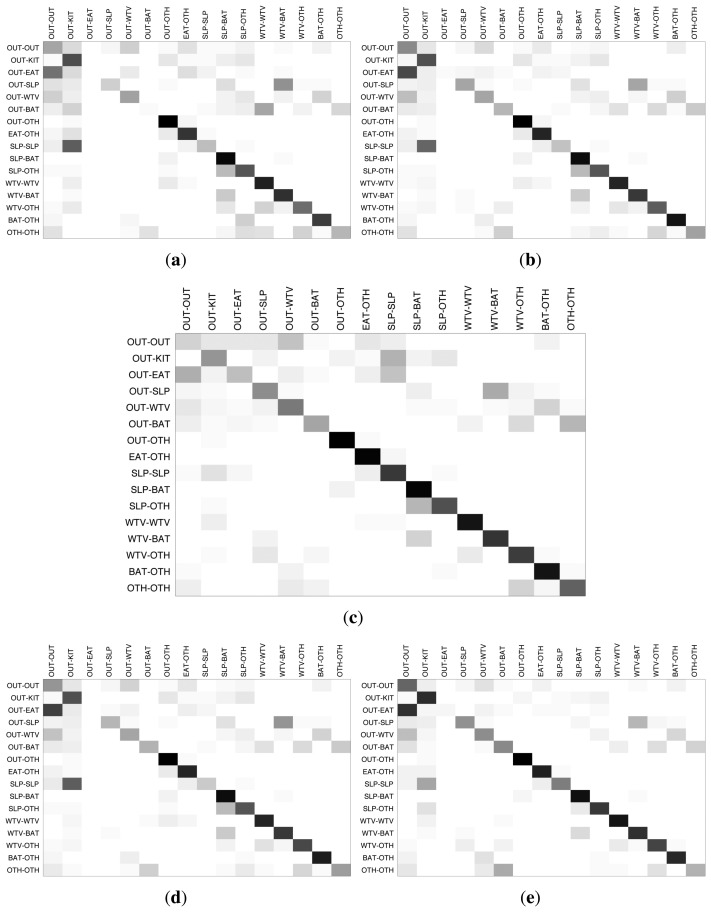
Confusion matrices for classification results of House A. (**a**) k-nearest neighbors (kNN); (**b**) decision tree (DT); (**c**) hidden Markov model (HMM); (**d**) multi-layer perceptron (MLP); (**e**) time-delay neural network (TDNN).

**Figure 10.**
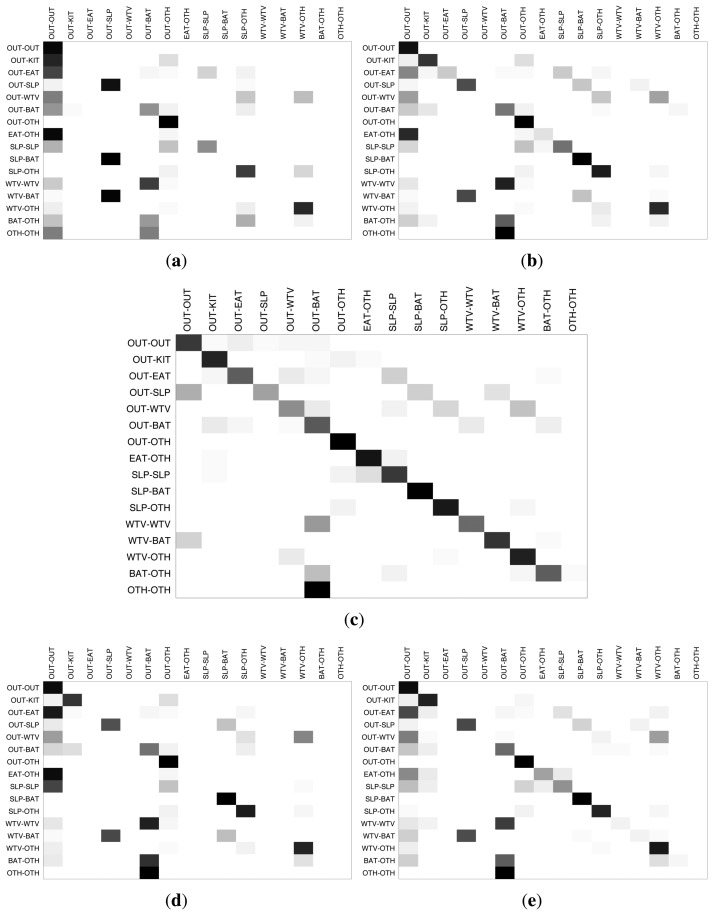
Confusion matrices for classification results of House B. (**a**) kNN; (**b**) DT; (**c**) HMM; (**d**) MLP; (**e**) TDNN.

In order to have a detailed look at the activity-wise inference performance of the classifiers, we provide the inter-activity confusion matrices for House A and House B in Figures 9 and 10, respectively. The darkness of the entries in the confusion matrices indicate the percentage of the estimations with respect to the ground-truth, with the ground-truth activities displayed on the left side and the estimated activities on the top side. The darker colors on the diagonals from the upper left to lower right indicate higher matching between the inferred activity and the ground-truth. For House A, HMM and TDNN, which are sequential methods, perform better than the other three classifiers. For House B, HMM provides fairly good results, which are consistent through most of the activities, while the other classifiers confuse some key activities.

In general, the most accurate classifications are obtained when one of the residents is not at home. This is expected due to the challenges stemming from the overlaid sensor readings. Secondly, the “other” activity category that contains several activities that are not performed every day, such as doing cleaning, having a guest and hanging out laundry, tends to be confused with the major activity categories, like eating, watching TV and bathroom activities. The most prominent example of such interference is the activity combination in which one resident is eating and the other one is engaged in some other activity. For instance, in House A, the residents have their meals on the table in the living room, and the same table is used also for studying, using the computer and many other activities. Therefore, it is very challenging for the classifiers to differentiate between the activities concerning the same household items.

**Figure 11.**
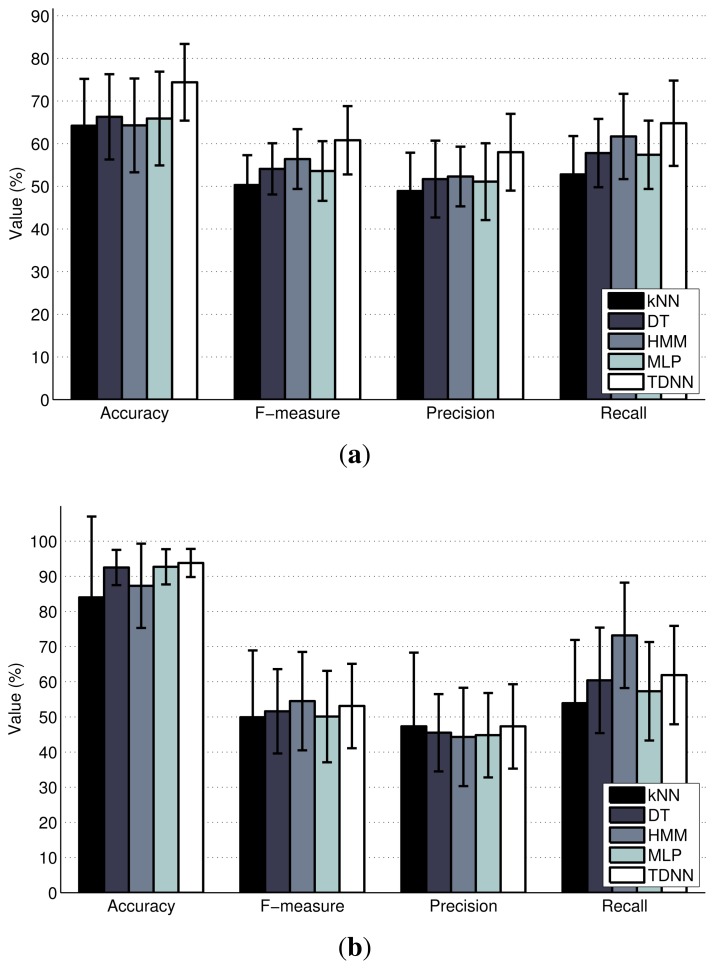
Activity classification results in terms of standard metrics. (**a**) House A; (**b**) House B.

We present the classifier performances in terms of the accuracy, F-measure, precision and recall metrics in Figure 11. These metrics support the interpretations made from the confusion matrices, as TDNN and HMM yield slightly superior performances compared to the other classifiers. The accuracy of these methods are also considerably good for these preliminary experiments considering the challenges related to inferring the activities of multiple residents.

### 5.3. Activity Recognition Individually

While it is possible to recognize the activities in a smart home without differentiating the residents, as described in the previous subsection, it is also possible to identify individual activities. This personalization enables more precise monitoring of residents' behaviors. For this purpose, we also performed individual activity recognition on our data sets. Tables 6 and 7 demonstrate the recognition performance of each activity in House A for the first and the second residents, respectively. According to the results, the overall recognition performance for the first resident is higher than the second resident. This difference stems from the training data sizes for the two residents. Since the second resident works outside longer hours, the training data for the activities inside the house becomes smaller; so, the models are not trained as well as the first resident's case. For both residents, the TDNN method consistently yields the best performance in terms of accuracy and the F-measure. In terms of activities, the sleeping activity is the best recognized activity and the eating activity is the most challenging one.

In Tables 8 and 9, we give the recognition performance in House B for the first and the second residents, respectively. The results shows that there is no significant difference in terms of overall recognition performance between residents. In this data set, the second resident has recorded no meal preparation activities; therefore, the KIT (kitchen) activity is omitted from the results for the second resident. As with House A, the sleeping activity is recognized with the highest accuracy and F-measure for House B. However, the bathroom activities are recognized with the lowest performance, although the metrics are higher than the eating activity in House A. The overall recognition performance in House B is greater than that of House A. The TDNN method gives the best performance for all houses and for all residents, consistently making it a suitable candidate for activity recognition.

## 6. Conclusion

Despite the challenges of processing large amounts of sensor data, wireless networking and the limitations of the sensor devices, WSNs are gradually being employed in the AAL domain. In this paper, we introduced an example multimodal WSN-based AAL system compatible for homes with multiple residents with the aim of recognizing the daily activities and routines of the users to detect the drifts and differences in their behavior, especially for monitoring their health and wellbeing status. In particular, we focused on the details of the system architecture and provided guidelines for the design and deployment of an effective AAL system. We presented the details of a field study to evaluate the success of the system, where it was deployed in two different real home environments with multiple residents and collected data from different types of ambient sensors about different activities for 30 full days. We discussed the possible applications for where this data can be used to assess the wellbeing of the residents and to identify deviations from everyday routines. Finally, we provided the results of several classification algorithms to recognize the activities of users from the sensor data. As future works, we will focus on the recognition performance to improve it even further with more sophisticated methods, like hierarchical factorial hidden Markov models. Secondly, we will focus on the scalability issues arising when these systems are to be deployed in a large scale.

## Figures and Tables

**Table 1.** General sensor selection criteria for house environments. (FSR: force sensitive resistor)

Sensor Type	Location	Targeted Activity	Robustness	Efficiency
FSR	Under bed	Lying, sleeping	High	High
	Under couch	Sitting, lying	High	High
	Under chair	Sitting	Low	Medium

Photocell	In drawer	Kitchen activities	High	High
	Cupboard/Wardrobe doors	Bathroom activities, changing clothes	High	High

Digital distance	Back of chair	Sitting	Medium	High
	Toilet seat cover	Bathroom activities	Medium	High
	Above water tap	Bathroom/kitchen activities	Medium	Medium

Sonar distance	Walls	Activity related to presence in a room	High	Medium

Contact	Regular door	Activity related to leave/entering room/house, showering	High	High
	Sliding door	Showering, changing clothes	Medium	Medium
	Drawer	Bathroom/kitchen activities	Low	Medium

Temperature	Above oven	Cooking	High	Medium
	Near stove	Cooking	Medium	Low

Infrared	Around TV	Watching TV	High	Medium

Humidity	Near shower sink	Showering	Medium	Low

Pressure Mat	On bed	Lying, sleeping	Medium	High
	On couch	Sitting, lying	High	High
	On chair	Sitting	Medium	Medium

Vibration	In drawer	Kitchen activities	Medium	Low

**Table 2.** Sensor battery lifetime experiment results.

		Sampling Rate	
		10 Hz	100 Hz
**Duty Cycle**	No duty cycling	24 h 10 min	24 h 02 min
	50%	49 h 23 min	45 h 32 min
	10%	128 h 21 min	120 h 43 min

**Table 3.** Properties of the datasets. PAN, personal area network.

	House A	House B
# of PANs	2	1
# of Ambient Sensors	20 of 7 different types	20 of 6 different types
Size of the House	50 m^2^	90 m^2^
House Information	One bedroom, one living room, one kitchen, one bathroom	2 bedrooms, one living room, one kitchen, one bathroom
Residents	2 males both aged 25	Married couple, age average 34
Duration	30 full days	30 full days
# of Activities	27	27

**Table 4.** Availability and locations of sensors in both houses.

Sensor	Location	House A	House B
Contact sensor on shower cabinet	Bathroom	✓	✓
Distance sensor above tap	Bathroom	✓	✓
Contact/distance sensor on door	Bathroom	✓	✓
Distance sensor on WC	Bathroom	✓	✓
Photocell in bathroom cabinet	Bathroom	✓	
Photocell in fridge	Kitchen	✓	✓
Photocell in drawer	Kitchen	✓	✓
Distance sensor on wall	Kitchen	✓	✓
Temperature sensor above oven	Kitchen	✓	
Contact sensor on right cupboard	Kitchen		✓
Contact sensor on left cupboard	Kitchen		✓
Force sensor on chair	Kitchen		✓
Force sensor on chair	Living room	✓	✓
Force sensor on chair	Living room	✓	✓
Infrared reader below TV	Living room	✓	
Force sensor on chair	Living room	✓	✓
Force sensor on armchair	Living room		✓
Force sensor on couch/bed	Living room/bedroom	✓	✓
Contact sensor/photocell in wardrobe	Living room/bedroom	✓	✓
Photocell in convertible couch	Living room/bedroom	✓	
Contact sensor/photocell in wardrobe	Bedroom	✓	✓
Force sensor on bed	Bedroom	✓	✓
Force sensor on bed	Bedroom		✓
Contact sensor on outside door	Hall	✓	✓
Distance sensor on wall	Hall	✓	

**Table 5.** Activity classes for grouping the activities in the datasets.

Abbreviation	Activity
OUT	Being Outside
SLP	Sleeping
KIT	Preparing a meal, washing dishes
EAT	Having a meal or snack
BAT	Toileting, having a shower, brushing teeth
WTV	Watching TV
OTH	All other activities performed in the house

**Table 6.** Activity recognition performance in House A for Resident 1.

	HMM		MLP		TDNN		kNN		DT	
	Acc	F-Measure	Acc	F-Measure	Acc	F-Measure	Acc	F-Measure	Acc	F-Measure
OTH	86.3	54.6	75.8	71.8	75.8	77.4	73.7	68.4	76.5	71.7
OUT	75.0	80.0	81.2	82.3	92.9	88.7	63.4	72.0	80.0	81.8
KIT	60.4	66.2	64.0	70.8	74.1	78.1	63.5	67.7	62.9	69.5
EAT	26.8	39.3	46.9	33.9	61.8	53.0	48.9	30.4	47.0	36.7
SLP	99.9	99.9	99.5	99.7	99.7	99.7	99.6	99.5	99.5	99.7
WTV	57.2	66.5	61.4	69.2	67.7	69.3	54.6	58.2	60.5	68.3
BAT	66.7	65.3	77.0	75.1	76.0	75.8	74.6	73.5	77.9	75.2

Average	67.5	67.4	72.3	71.8	78.3	77.4	68.3	67.1	72.0	71.8

**Table 7.** Activity recognition performance in House A for Resident 2.

	HMM		MLP		TDNN		kNN		DT	
	Acc	F-Measure	Acc	F-Measure	Acc	F-Measure	Acc	F-Measure	Acc	F-Measure
OTH	47.6	39.1	49.9	29.6	54.4	42.0	49.3	34.0	47.9	33.1
OUT	89.0	90.2	81.1	88.5	87.1	91.6	76.5	83.8	81.6	88.4
KIT	32.1	41.4	0.0	0.0	50.8	31.8	14.6	1.9	20.2	6.9
EAT	36.8	43.4	44.4	40.6	51.0	48.0	53.5	17.2	42.6	44.4
SLP	82.7	77.8	86.7	81.6	87.4	85.1	88.5	81.0	86.9	80.6
WTV	53.8	58.2	54.6	57.4	56.2	58.7	41.8	46.8	54.4	55.4
BAT	55.6	62.6	78.2	65.8	70.2	67.7	87.4	54.9	75.0	65.4

Average	56.8	58.9	56.4	51.9	65.3	60.7	58.8	45.7	58.4	53.4

**Table 8.** Activity recognition performance in House B for Resident 1.

	HMM		MLP		TDNN		kNN		DT	
	Acc	F-Measure	Acc	F-Measure	Acc	F-Measure	Acc	F-Measure	Acc	F-Measure
OTH	94.7	83.2	88.2	83.9	90.9	89.1	86.3	83.2	91.6	85.1
OUT	97.9	98.9	96.1	98.0	98.3	99.0	96.1	98.0	96.2	98.0
KIT	70.5	79.7	58.8	55.3	73.1	76.2	52.8	48.7	61.2	67.4
EAT	78.0	80.4	94.6	85.1	87.7	81.8	97.0	81.8	95.7	85.3
SLP	99.9	99.8	99.8	99.9	99.6	99.8	99.6	99.8	99.8	99.9
WTV	82.0	87.8	88.3	89.3	90.9	91.1	87.8	88.9	89.1	89.7
BAT	46.3	53.4	51.2	40.6	68.7	59.9	61.9	36.4	63.5	59.7

Average	81.3	83.3	82.4	78.9	87.0	85.3	83.1	76.7	85.3	83.6

**Table 9.** Activity recognition performance in House B for Resident 2.

	HMM		MLP		TDNN		kNN		DT	
	Acc	F-Measure	Acc	F-Measure	Acc	F-Measure	Acc	F-Measure	Acc	F-Measure
OTH	87.3	76.8	81.1	73.6	80.7	78.3	41.9	55.6	82.8	74.5
OUT	96.2	97.7	94.4	96.2	96.4	97.4	95.2	87.1	93.7	96.1
EAT	96.4	94.8	80.6	80.1	92.0	85.7	98.7	83.5	97.7	89.5
SLP	99.9	99.9	99.8	99.7	99.7	99.8	99.8	99.7	99.8	99.9
WTV	82.6	83.8	82.4	80.7	83.6	83.3	82.8	79.3	84.6	80.2
BAT	45.4	52.0	51.7	46.8	70.8	55.1	57.1	38.1	65.6	50.7

Average	84.6	84.2	81.7	79.5	87.2	83.3	79.2	73.9	87.4	81.8
